# Differential White Matter Connectivity in Early Mild Cognitive Impairment According to CSF Biomarkers

**DOI:** 10.1371/journal.pone.0091400

**Published:** 2014-03-10

**Authors:** Jae-Sung Lim, Young Ho Park, Jae-Won Jang, So Yong Park, SangYun Kim, for the Alzheimer's Disease Neuroimaging Initiative

**Affiliations:** 1 Department of Neurology, Seoul Metropolitan Government-Seoul National University Boramae Medical Center, Seoul, Republic of Korea; 2 Clinical Neuroscience Center, Seoul National University Bundang Hospital, Seongnam, Republic of Korea; 3 Department of Neurology, Seoul National University College of Medicine, Seoul, Republic of Korea; University of Manchester, United Kingdom

## Abstract

Mild cognitive impairment (MCI) is a heterogeneous group and certain MCI subsets eventually convert to dementia. Cerebrospinal fluid (CSF) biomarkers are known to predict this conversion. We sought evidence for the differences in white matter connectivity between early amnestic MCI (EMCI) subgroups according to a CSF phosphorylated tau_181p_/amyloid beta_1–42_ ratio of 0.10. From the Alzheimer's Disease Neuroimaging Initiative database, 16 high-ratio, 25 low-ratio EMCI patients, and 20 normal controls with diffusion tensor images and CSF profiles were included. Compared to the high-ratio group, radial diffusivity significantly increased in both sides of the corpus callosum and the superior and inferior longitudinal fasciculus in the low-ratio group. In widespread white matter skeleton regions, the low-ratio group showed significantly increased mean, axial, and radial diffusivity compared to normal controls. However, the high-ratio group showed no differences when compared to the normal group. In conclusion, our study revealed that there were significant differences in white matter connectivity between EMCI subgroups according to CSF phosphorylated tau_181p_/amyloid beta_1–42_ratios.

## Introduction

Mild cognitive impairment (MCI) is a heterogeneous group, and certain subsets eventually convert to dementia [1]. In a new classification by the National Institute of Aging-Alzheimer's Association (NIA-AA), MCI is classified as MCI due to Alzheimer's disease (AD) or unlikely due to AD based on multiple biomarkers, as well as core clinical features suggesting AD [2], [3].

Cerebrospinal fluid (CSF) Amyloid beta peptides _1–42_ (Aβ_1–42_) are considered as an early indicator of AD conversion compared to CSF tau [4]. Though decreased CSF Aβ_1–42_ levels usually accompany high-tau groups, Aβ_1–42_ levels may show variable concentrations even with similar tau levels, especially in early amnestic MCI or stable MCI [5], [6]. Considering that there are heterogeneous subsets in MCI, this variability brought up questions about grouping MCI based on absolute values of CSF total tau, even with age-attributed norm data [7], [8]. Thus we thought both CSF Aβ_1–42_ and tau should be simultaneously considered to optimally classify MCI subgroups.

CSF tau phosphorylated at threonine 181/Aβ_1–42_ ratios (pTau/Aβ ratio) were proposed as one of the best predictors to discriminate AD conversion groups from non-conversion group; with a sensitivity of 81–88% and a specificity of 90–95% [4], [6]. A ratio with a threshold value of 0.10 was put forth as a useful marker for a differential diagnosis of AD vs. normal subjects; with 91.1% sensitivity and 71.2% specificity [5].

Diffusion tensor images (DTI) recently gained attention because of its detection of white matter disintegration in various neurological disorders. In addition to classical parameters such as fractional anisotropy (FA) and mean diffusivity (MD), other indices such as axial diffusivity (DA) and radial diffusivity (DR) also revealed significant changes in MCI and AD patients [9]. However, DTI has been classified as a ‘less well validated biomarker’ in the NIA-AA classification system [2].

Until now, few reports have investigated white matter microstructural differences between MCI subtypes defined by both CSF amyloid and tau levels. Therefore, we sought evidence for white matter structural differences between early MCI subgroups according to the pTau/Aβ ratio. Additionally, we investigated amyloid positivity in each MCI group using Florbetapir PET.

## Methods

### Ethics Statement

The institutional review board of Seoul National University Bundang Hospital approved this study. Detailed protocols for informed consent of Alzheimer's Disease Neuroimaging Initiative (ADNI) subjects can be referenced in ADNI information pages. (www.adni-info.org.).

### Database and Subjects

The detailed explanation for the ADNI was described in supplemental methods. For up-to-date information, see www.adni-info.org.

In January 2013, we queried early MCI (EMCI) and normal subjects from the ADNI database (https://ida.loni.ucla.edu/). ADNI had begun to enroll EMCI subjects in an effort to incorporate the mildest symptomatic phase of dementia. The inclusion and exclusion criteria for EMCI are described on the ADNI website [10], [11]. For details, subjects were required to have either subjective or objective memory problems. Abnormal memory function was determined by scoring below the education-adjusted cutoff on the Logical Memory II subscale (Delayed Paragraph Recall) from the Wechsler Memory Scale – Revised (between approximately 0.5 and 1.5 standard deviation below the mean of Cognitively Normal). Mini-Mental State Exam scores needed to be between 24 and 30 (inclusive), and a clinical dementia rating of 0.5. Additionally, it was required that other cognitive domains and functional performance be sufficiently preserved. Patients who had any significant neurologic diseases such as brain tumor, seizure disorder, multiple sclerosis were excluded.

We collected subject's baseline CSF data, DTI images, and Florbetapir positron emission tomography (PET) from the ADNI database (https://ida.loni.ucla.edu/). If patients had multiple DTI images, our analysis only included the initial screening images from when baseline CSF drainage was conducted. We excluded subjects with any structural abnormalities such as old lacunar infarctions or severe white matter hyperintensities in non-diffusion weighted images.

### CSF Biomarkers and Grouping of Study Subjects

Detailed protocols for CSF collection and analysis have been previously published [5]. CSF samples were obtained from study subjects in the early morning after overnight fasting. Samples were then transferred to the ADNI Biomarker Core laboratory at the University of Pennsylvania Medical Center, and were subsequently analyzed to determine Aβ_1–42_ and pTau_181p_ concentrations using the multiplex xMAPLuminex platform (Luminex) with Innogenetics (INNO-BIA AlzBio3) immunoassay kit–based reagents [5].

For further analysis, we grouped the above subjects according to the CSF pTau/Aβ ratio with a cut-off value of 0.10, and subdivided them into high- and low-ratio groups [5].

### Diffusion Tensor Images and Image processing

Specific DTI protocols can be found on the ADNI website (http://adni.loni.ucla.edu/methods/documents/mri-protocols/). In brief, ADNI investigators conducted DTI using 3-Tesla General Electric Healthcare scanners. Detailed protocols were as follows: 256×256 matrix; voxel size: 1.4×1.4×2.7 mm^3^; scan time  = 9 min; 5 T2-weighted images with no diffusion sensitization (b0 images); 41 diffusion-weighted images (b = 1000 s/mm^2^). Only non-processed, original axial DTI images were downloaded as archived formats from the ADNI Image Data Archive website (https://ida.loni.ucla.edu/).

For DTI preprocessing, all images were skull-stripped and brain extracted using the *Brain Extraction Tool*, embedded in FSL (version 4.1.8, FMRIB) [12], [13]. Then the terminal command, *eddy_correct*, was used for eddy current correction. Each DTI map – FA, MD, DA – was constructed based on calculated tensors using *DTIFIT*, an embedded tool in the FSL library. DR was calculated as the mean of the second and third eigenvalues ((λ2+λ3)/2) using the terminal command, *fslmaths*.

We used tract-based spatial statistics (TBSS) for further analysis [14]. Native FA data was non-linearly registered to common templates (FMRIB58_FA_1 mm), and then projected to a pre-calculated FA skeleton based on FA data from study subjects. Thereafter, a FA threshold of 0.2 was applied to the skeletons to reduce erroneous inclusion of non-white matter tissues and misalignment issues in further analysis. For MD, DA, and DR maps analysis, we applied the same transformation process used in FA maps.

Voxelwise statistics were conducted for all skeletonized FA data. *Randomise*, an embedded tool for voxelwise statistical analysis, was used for further statistical analysis with threshold-free cluster enhancement methods and 5000 permutations [15]. Statistical significance was set at p<0.05 with multiple comparison correction using a family wise error comparison. Using fslview, results were mounted on standard 1 mm T1 images (MNI152_T1_1 mm_brain) in the FSL library. Detailed white matter skeleton nomenclatures were identified using the JHU white-matter tractography atlas, also embedded in the FSL library.

### Florbetapir PET

For underlying amyloid pathology investigation, we identified the Florbetapir mean standard uptake value ratio (SUVR) for each group. Detailed protocols for Florbetapir PET acquisition and processing can be found on the ADNI website (http://adni.loni.ucla.edu/methods/documents; ADNI_UC_Berkeley_AV45_Methods_20121026.pdf, https://ida.loni.ucla.edu/). UC Berkeley and Lawrence Berkeley National Laboratory conducted the entire Florbetapir data analysis, and we used this dataset (UC Berkeley – AV45 analysis). In brief, cortical grey matter regions of interest (frontal, anterior/posterior cingulated, lateral parietal, lateral temporal) and the reference region (whole cerebellum) were defined with a native-space magnetic resonance imaging (MRI) scan for each subject using Freesurfer (version 4.5.0). Florbetapir PET images were co-registered with the corresponding MRI; then mean Florbetapir uptake values were calculated for each cortical and reference region. To determine the SUVR for each subject, the average of the four cortical regions uptake values was divided by the reference region uptake value. The amyloid positivity cutoff value was set at 1.11 according to the recommendation of the UC Berkeley analysis group.

### Statistical analysis

Continuous variables such as age, education level, and mini-mental status examination scores were compared using one-way analysis of variance with Bonferroni's post hoc multiple comparisons. Categorical variables – gender and apolipoprotein E (ApoE) genotyping were analyzed using Chi-square or Fisher's Exact test. Any unbalanced variables among groups were included in *Randomise* design matrix to regress out the covariate confounding effects in TBSS analysis [15] [16].

## Results

**Figure 1:**
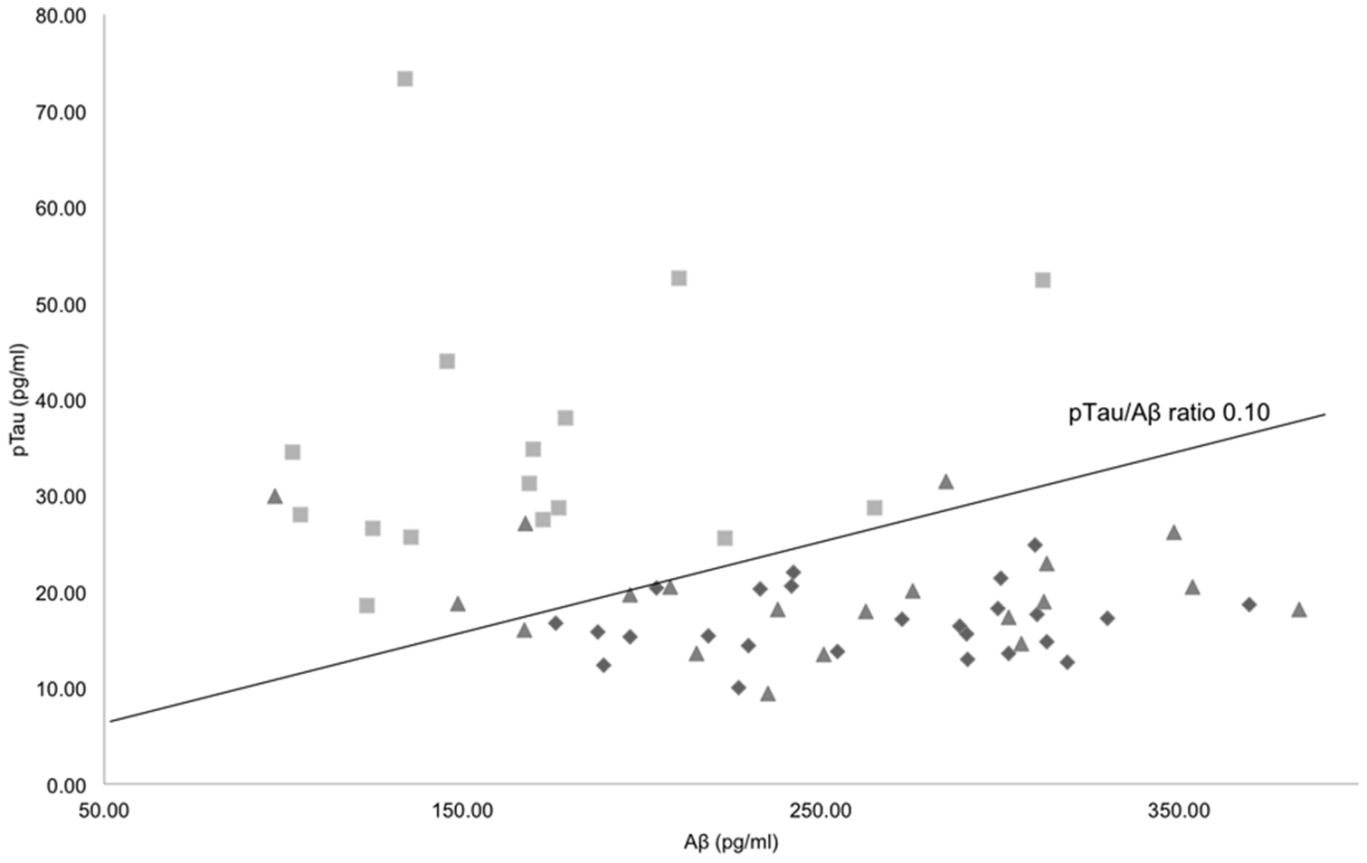
Scatter plots of CSF Aβ versus pTau concentrations for normal controls (triangles), low-ratio (diamonds), and high-ratio (rectangles) EMCI groups. Linear plot lines denote the CSF pTau/Aβ ratio, 0.10. Abbreviations: Aβ (amyloid beta_1–42_), pTau (phosphorylated tau at threonine 181).

After excluding 6 subjects with old lacunar infarction or significant white matter hyperintensities, a total of 61 EMCI and normal subjects (16 high-ratio, 25 low-ratio, 20 normal controls) with DTI and CSF profiles were included from the ADNI database (Figure 1).

Education levels were different between the high versus low pTau/Aβ ratio EMCI groups (Table 1). However, there were no significant differences in other clinical variables, including ApoE4 genotype. ApoE2 genotypes were observed in only 2 patients. To regress out the confounding effect of baseline characteristics in the following analyses, we included demeaned age and education levels of each subject into the design matrix in the *randomize* tool of fsl.

**Table 1:** Baseline Characteristics of Study Subjects.

	Normal (n = 20)	Low-ratio (n = 25)	High-ratio (n = 16)	*p*-value*
Age (years)	74.75±5.26	71.52±7.38	74.25±7.96	0.25
Male	11 (55.0%)	17 (68.0%)	8 (50.0%)	0.47
Education (years)	16.25±2.40	17.12±2.09	14.50±2.73	< 0.01
T†	a, b	a	b	
MMSE	28.90±1.62	28.20±1.44	27.44±1.79	0.03
T†	a	a, b	b	
ApoE ε4 carriers	4 (20%)	7 (28.0%)	6 (37.5%)	0.55‡

Figures denote mean values and standard deviations. Parentheses indicate frequencies.

^*^ Statistical significances were tested by one-way analysis of variances among groups.

† The same letters indicate non-significant difference between groups based on Bonferroni multiple comparison tests.

‡ Fisher's Exact Test.

Abbreviations: MMSE (Mini-Mental State Examination).

**Figure 2:**
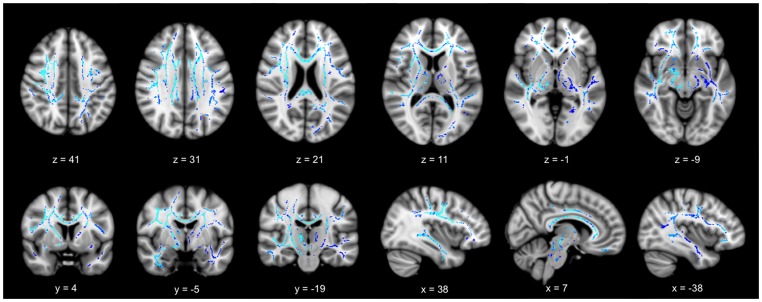
Tract-based spatial statistics results between high versus low-ratio groups (corrected *p*-value <0.05). Radial diffusivity of corpus callosum, superior and inferior longitudinal fasciculus, and anterior thalamic radiation increased in the low-ratio group (blue color). The light blue color indicates more significant changes compared to dark blue. Each number below images indicates MNI coordinates of corresponding template sections.

In corrected images for multiple comparisons, we found that DR of the low-ratio group was significantly increased in both sides of the corpus callosum, forceps minor, and superior and inferior longitudinal fasciculus after adjusting for covariates compared to the high-ratio group (Figure 2). However, FA, MD, and DA did not reveal any significant differences between the two EMCI groups. When we applied a more conservative corrected *p*-value (corrected *p*-value <0.03) to better discriminate between groups, the differences between high- and low-ratio groups were more prominent in frontotemporal white matter than parieto-occipital regions (Figure S1).

**Figure 3:**
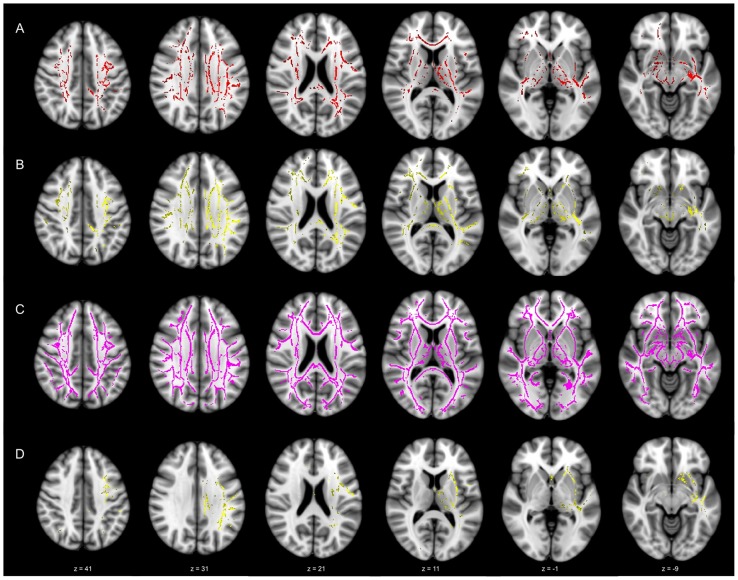
Tract-based spatial statistics results between normal controls versus low-ratio (A–C) and high-ratio (D) groups. Mean, axial, and radial diffusivity of widespread white matter skeletons increased in the low-ratio group (A–C; red, yellow, pink, respectively). Axial diffusivity of left-sided superior longitudinal fasciculus, external capsule, anterior thalamic radiation, cingulum, and inferior fronto-occipital fasciculus increased in the high-ratio group (D, yellow, uncorrected data). Each number below the column denotes MNI coordinates of corresponding template sections.

When we compared the low pTau/Aβ ratio EMCI groups with normal subjects, the low-ratio group showed significantly increased MD, DA, and DR changes in widespread white matter skeleton regions after adjusting ages (Figure 3A–C). DR maps showed the most extensive distribution differences among the above three DTI indices (Figure 3C). However, no differences were seen between the high-ratio group and the normal group, though there was a tendency of increased DA in the left superior longitudinal fasciculus, cingulum, external capsule, anterior thalamic radiation, and inferior fronto-occipital fasciculus in the high-ratio group (uncorrected *p*-value 0.014, corrected *p*-value 0.156) (Figure 3D).

In post-hoc analysis, 25% of subjects in normal group showed amyloid positivity. Thus, for an exact comparison, the normal control group was redefined based on biomarkers as well as clinical characteristics. Only those who met the criteria where baseline CSF pTau/Aβ ratio was no more than 0.10, CSF Aβ no less than 192 pg/ml, and Florbetapir SUVR less than 1.11 among normal controls in ADNI database were included for this analysis [5] (http://adni.loni.ucla.edu/methods/documents; ADNI_UC_Berkeley_AV45_Methods_20121026.pdf, https://ida.loni.ucla.edu/). The baseline characteristics of this redefined normal group were presented in supplementary material (Table S1–2).

When we compared the low pTau/Aβ ratio EMCI groups with redefined 13 normal subjects, the low-ratio group showed significantly increased MD, DA, and DR changes in widespread white matter skeleton regions compared to normal group after adjusting for age (Figure S2). However, once again, the high-ratio group did not reveal any significant differences with the normal group.

The mean SUVR of the high-ratio group was significantly higher than those of the low-ratio and normal groups (1.33±0.21, 1.02±0.08, 1.06±0.16, respectively; *p*-value <0.01) (Table 2). The mean SUVR of redefined 13 normal subjects was 0.99±0.06. Amyloid positivity was significantly more frequent in the high-ratio group than in the low-ratio or normal groups (81.3% vs. 4.2% vs. 25.0%, respectively; *p*-value <0.01).

**Table 2:** CSF Biomarkers and Florbetapir Standard Uptake Value Ratios of Study Subjects.

	Normal (n = 20)	Low-ratio (n = 25)	High-ratio (n = 16)	*p*-value*
CSF biomarkers				
Aβ (pg/ml)	253.35±75.08	263.87±52.07	171.71±57.63	<0.01†
pTau (pg/ml)	19.72±5.56	16.72±3.49	35.63±13.85	<0.01†
Total Tau (pg/ml)	66.16±23.09	60.36±20.39	125.44±64.31	<0.01†
pTau/Aβ ratio	0.09±0.06	0.07±0.02	0.22±0.11	<0.01†
Florbetapir PET				
Mean SUVR	1.06±0.16	1.02±0.08	1.33±0.21	<0.01†

Figures denote mean values and standard deviations.

^*^ Statistical significances were tested by one-way analysis of variance among groups.

† Bonferroni multiple comparison tests show significant differences between high-ratio vs. low-ratio or high-ratio vs. normal group. There are no significant differences between low-ratio and normal groups.

Abbreviations: CSF (cerebrospinal fluid), Aβ (amyloid beta_1–42_), pTau (phosphorylated Tau_181p_), PET (positron emission tomography), SUVR (standard uptake value ratio).

When we analyze the correlation between CSF pTau/Aβ ratio and Florbetapir SUVR, there was significant positive correlation in EMCI groups (Table S3, Figure S3). This correlation was still significant after adjusting education levels (partial correlation coefficient 0.62, p-value <0.01). However, these associations were not detected in normal control group.

## Discussion

Among the EMCI subjects, our results revealed that there were significant differences in white matter integrities reflected by DR maps between high and low pTau/Aβ ratio EMCI groups. Compared to normal control, the low-ratio group showed significant DR increases in widespread regions and marginal increases of MD and DA along with decreases of FA, while the high-ratio group did not show any significant differences with normal control. Several explanations might be suggested for these findings.

### Aβ-independent neuronal injuries in low-ratio group

In our results, the low-ratio group might be regarded as ‘suspected non-AD pathophysiology (sNAP)’ who had neuronal injury biomarker without amyloid biomarker [17], [18]. In the recent cohort, they consisted of about 23% of elderly cognitively normal subjects [17]. Even if they were preclinical AD patients, their cognitive deficit might be due to Aβ-independent neuronal injuries considering low CSF pTau/Aβ ratio and negative Florbetapir PET [19]. In the recent paper, DTI changes might not necessarily reflect underlying amyloid pathology, and were suggested to be possibly through non-AD pathological cascade [19]. Thus, the neuronal injury biomarkers of the low-ratio group might not be dependent on Aβ.

On the other hand, the high-ratio group might be classified as ‘preclinical AD’ according to NIA-AA criteria considering CSF biomarkers signature in AD as well as Florbetapir positivity [2]. Considering the proposal of sequential biomarkers changes, they can be classified as stage 3 in which subtle cognitive impairment is evident, and the neuronal injury markers should be also evident in this stage [17], [20]. However, the high-ratio group was positive only in the amyloid biomarkers (Florbetapir PET and CSF ratio) and negative for neuronal injury markers (DTI). Although we did not investigate hippocampal atrophy or FDG-PET hypometabolism that is regarded as neuronal injury biomarkers, there were certain portions of preclinical AD patients who had amyloid biomarker but did not have neuronal injury biomarkers in the previous study [17]. Furthermore, there are some controversies for the proposal of sequential biomarkers changes [18].

While the former studies did not find any differences between sNAP and preclinical AD, they did not include DTI in the study variables [18]. DTI indicies, especially DR, were suggested to be the most sensitive parameters to detect the subtle differences in amnestic MCI compared to normal controls [21]–[23]. DR might reflect generally amyloid pathology in AD patients rather than intraaxonal tau pathology, however it is not specific or exclusive biomarker of amyloid [24]. DR changes indicate the extra-axonal pathologies such as demyelination along with loss of oligodendrocytes, glial cell activation, and extracellular inflammation [23]. Thus, the differences in DTI biomarkers between low- and high-ratio groups might be interpreted as the differences between sNAP and preclinical AD.

### Demographic factors, cognitive reserve, and brain plasticity

Several demographic factors should be also considered to interpret our results. Mean age of our normal control group was 75.6 years compared to 61.7 and 66 years of the previous studies [8], [9]. Considering that aging affects the white matter integrities including DR [25], aging might attenuate the already-known white matter structural differences between normal and MCI. There were also significant differences in education levels between high-ratio and low-ratio groups. There are no reports in the literature until now about education effect on DR. We included education levels into the design matrix in *randomize* tool of fsl for regressing out the confounding effect of differences in education [16].

Although the low-ratio group was younger, and well educated compared to high-ratio groups, (Table 1) they showed similar level of cognitive deficits, which suggested the possibility of more pronounced underlying structural changes coped with cognitive reserve [19]. Previous study revealed that cognitive reserve had negative association with fractional anisotropy in amnestic MCI patients, which might reflect increased capacity to cope with incipient cerebral damage [26].

Cholinesterase inhibitors might also confer the neurotrophic and neurorestorative effect in AD patients, which reflected by increased anatomical connectivity mapping [27]. In ADNI EMCI patients, cholinesterase inhibitors and memantine were allowable if stable for 12 weeks prior to screening [10]. However, only 5 patients of EMCI group had been prescribed with cholinesterase inhibitors at baseline. Thus, above-mentioned brain plasticity caused by cholinesterase inhibitors might not be properly analyzed in our study.

Another consideration is that our analysis was limited to very early stages of MCI. The discrimination between normal control groups and EMCI in ADNI 2 and ADNI-GO was only based on logical memory test, MMSE, and CDR. Furthermore, as we have stated in the results section, there were considerable number of subjects who showed amyloid positivity in the normal control group. This might be another reason why the normal control group failed to show significant differences compared to the high-ratio group, being considered as preclinical AD subjects. After excluding those who showed abnormalities in the biomarkers, high-ratio group did not show any significant differences with normal controls. However, small number of patients might also affect the analysis.

### Distribution of DTI indices abnormalities

The low-ratio group showed increased DR in both sides of the corpus callosum, forceps minor, anterior thalamic radiation, superior, and inferior longitudinal fasciculus compared to the high-ratio group. Long association fibers, such as superior and inferior longitudinal fasciculi, are late-myelinating fibers [28]. Previous studies with amnestic MCI patients revealed the abnormalities in DA, DR, and FA of these fibers [28], [29]. In a recent study, DR was increased in the high-CSF tau MCI group in the right superior and inferior longitudinal fasciculus, and in the hippocampal area of the cingulum [8]. In addition, MD was increased in the genu, splenium of corpus callosum, and both corona radiata [30]. However, most studies did not investigate amyloid deposition of those areas simultaneously.

Several large-scale functional networks such as default mode network (DMN) were disrupted in MCI and AD patients [31], [32]. In the early phase, the posterior white matter tracts, which connect the medial temporal structures and posterior cingulate cortex, were affected first, while the structural integrities of the anterior white matter tracts such as genu of corpus callosum were still maintained [33]. In our results, abnormalities in the low-ratio group were not restricted in the posterior and limbic white-matter tracts, but also distributed in the frontal regions. However, the structural abnormalities of the large-scale functional networks should be considered to interpret our results.

### Correlation between CSF pTau/Aβ ratio and Florbetapir SUVR

Another finding in our results was the correlation between CSF biomarkers and Florbetapir SUVR. One recent study showed a reverse correlation between CSF Aβ_1–42_ and Florbetapir in AD, MCI, and normal subgroups of ADNI DB [34]. In our results, mean SUVR of the high-ratio EMCI group was significantly higher than those of low-ratio EMCI and normal control group. CSF Aβ_1–42_ biomarkers were negatively correlated with Florbetapir SUVR only in MCI stages, which reflect that decreasing Aβ in MCI might suggest the accumulation of Aβ in cortical neuritic plaques. On the contrary, these associations might not be applicable to the normal controls.

### Study Limitations

There are some limitations in this study. First, due to a cross-sectional study design, which did not allow follow-up data, we could not determine the conversion to AD or other types of dementias with our EMCI subgroups. Second, because of the limited number of subjects in each group, these results should be regarded as preliminary. Further longitudinal studies are needed to confirm our results. Third, tau is not necessarily phosphorylated at threonine 181. We adopted pTau_181_/Aβ_1–42_ based on the previous studies. However, there are evidences for other phosphorylation sites for tau protein could be helpful to discriminate AD with non-AD dementia [35]. For examples, pTau phosphorylated at threonine 231 was useful to differentiate AD and FTD. Phosphorylated tau at serine 306/serine 404 could differentiate between AD and vascular dementia. Phosphorylated tau at threonine 181 was helpful to differentiate AD and DLB. Thus, though high predictive power of pTau_181_/Aβ_1–42_ for AD conversion, we should consider limitation of CSF pTau_181_/Aβ_1–42_ to classify subgroups of EMCI. Fourth, ApoE genotypes should be considered in these kinds of structural imaging studies with regard to MCI or AD. Widespread differences in DTI parameters according to ApoE genotypes were recently reported using the ADNI database [36]. In their results, ApoE ε2 carriers revealed higher FA values in widespread regions such as in the superior longitudinal fasciculus, right thalamus, both anterior limbs of internal capsules, posterior cingulum, and corpus callosum [36]. In the above-mentioned regions, we identified a partial overlap with our results. However, in our study, ApoE2 genotypes were observed in only 2 patients, we could not state the effect of ApoE2 in our analysis. Lastly, we conducted TBSS analysis, which is hypothesis-free technique. We thought that TBSS analysis was more appropriate to avoid any possible bias during tractography. However, statistical significance by TFCE method – a fully corrected for multiple comparisons across space – was rather conservative for small number of subjects, and this methodological issue might be one of reasons why we could not find any significant differences between the high-ratio and normal control group.

### Clinical implications

There were continuing efforts to discriminate the subtypes of MCI for early detection of prodrome of various neurodegenerative dementias [2]. Clinical classifications, which specify amnestic/non-amnestic, single-domain/multiple-domains, had limitations to predict the exact conversion of specific neurodegenerative dementias [37]. Thus, recent studies incorporated neuroimaging biomarkers to predict these conversions in MCI stages [38].

Our study showed that white matter structures revealed significant differences according to CSF biomarkers in early stage of MCI. This suggests that there may be distinctive white matter structural differences in early stage MCI subgroups, and support the previous studies that classified MCI subjects according to CSF biomarkers. However, the exact pathological meanings of increased DR in low-ratio group remain to be validated by further studies.

In conclusion, our study suggests that there are significant white matter integrity differences between EMCI subtypes according to CSF pTau/Aβ ratios. Furthermore, DTI biomarkers may reflect Aβ-independent neuronal injuries in EMCI patients.

## Supporting Information

Figure S1 **Tract-based spatial statistics results between high versus low-ratio groups (corrected** ***p*** **-value <0.03).** Radial diffusivity of the corpus callosum and the right superior and inferior longitudinal fasciculus increased in the low-ratio group (Blue). A white matter skeleton (in green) was created using mean fractional anisotropy, derived from both groups. For better visualization of any significant results, we used a *tbss_fill* – fsl terminal command- with a threshold corrected *p*-value <0.03. Numbers below images denote MNI coordinates of corresponding template sections.
(TIF)

Figure S2 **Tract-based spatial statistics results between redefined normal controls versus low-ratio (A–C) groups adjusting for age.** Mean, axial, and radial diffusivity of widespread white matter skeletons increased in the low-ratio group (A–C; red, yellow, pink, respectively). Each number below the column denotes MNI coordinates of corresponding template sections.
(TIF)

Figure S3 **Scatter plot for CSF pTau/Aβ ratio and Florbetapir SUVR in EMCI subjects.** Scatter plot showed correlation between CSF pTau/Aβ ratio and Florbetapir SUVR in EMCI subjects.
(TIFF)

Table S1 **Baseline Characteristics of Redefined Study Subjects.**
(DOCX)

Table S2 **CSF Biomarkers and Florbetapir Standard Uptake Value Ratios of Study Subjects.**
(DOCX)

Table S3 **Correlation coefficient (ρ) between CSF biomarkers and Florbetapir SUVR.**
(DOCX)

File S1 **Supplemental Methods.**
(DOCX)
