# Supplementary figures and images for: Differential White Matter Connectivity in Early Mild Cognitive Impairment According to CSF Biomarkers
Source: PLoS One. 2014 Mar 10;9(3):e91400. doi: 10.1371/journal.pone.0091400 (PMC3948821; doi:10.1371/journal.pone.0091400)

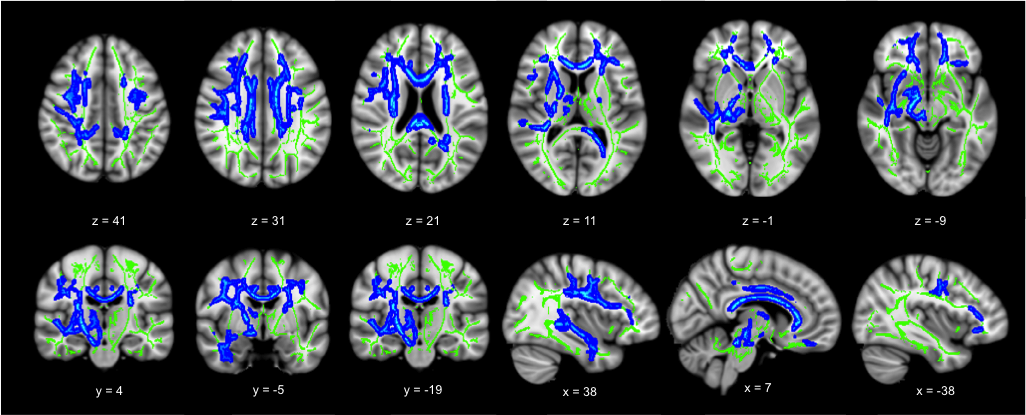

Supplement: Figure S1 — Tract-based spatial statistics results between high versus low-ratio groups (corrected p -value <0.03). Radial diffusivity of the corpus callosum and the right superior and inferior longitudinal fasciculus increased in the low-ratio group (Blue). A white matter skeleton (in green) was created using mean fractional anisotropy, derived from both groups. For better visualization of any significant results, we used a tbss_fill – fsl terminal command- with a threshold corrected p-value <0.03. Numbers below images denote MNI coordinates of corresponding template sections. (TIF) [file pone.0091400.s001.tif]

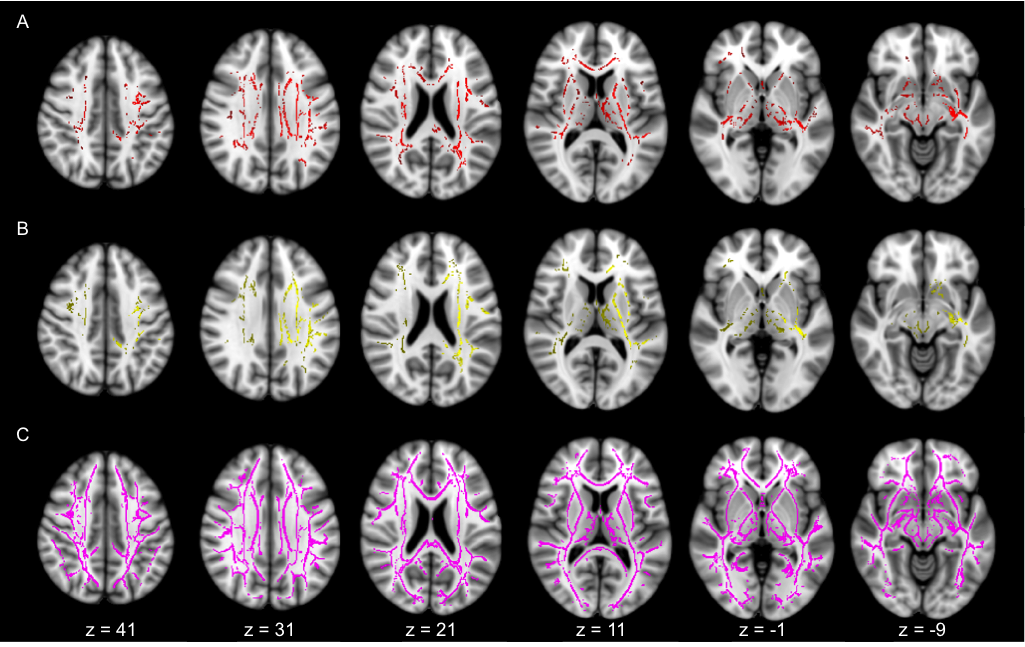

Supplement: Figure S2 — Tract-based spatial statistics results between redefined normal controls versus low-ratio (A–C) groups adjusting for age. Mean, axial, and radial diffusivity of widespread white matter skeletons increased in the low-ratio group (A–C; red, yellow, pink, respectively). Each number below the column denotes MNI coordinates of corresponding template sections. (TIF) [file pone.0091400.s002.tif]

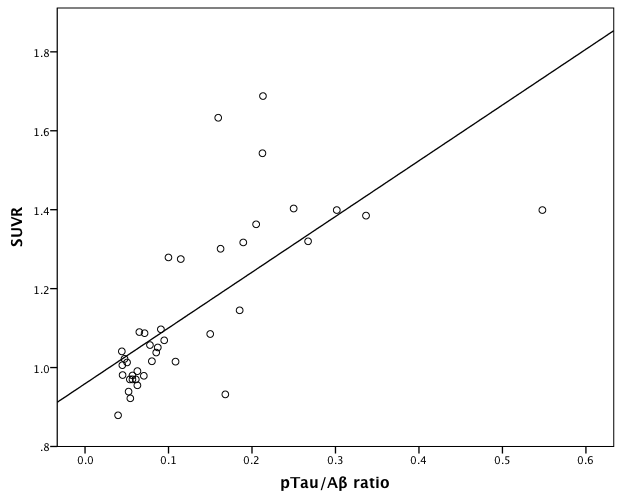

Supplement: Figure S3 — Scatter plot for CSF pTau/Aβ ratio and Florbetapir SUVR in EMCI subjects. Scatter plot showed correlation between CSF pTau/Aβ ratio and Florbetapir SUVR in EMCI subjects. (TIFF) [file pone.0091400.s003.tiff]
