# Supplementary material for: Differential White Matter Connectivity in Early Mild Cognitive Impairment According to CSF Biomarkers
Source: PLoS One. 2014 Mar 10;9(3):e91400. doi: 10.1371/journal.pone.0091400 (PMC3948821; doi:10.1371/journal.pone.0091400)
Supplement: Table S1 — Baseline Characteristics of Redefined Study Subjects. (DOCX) [file pone.0091400.s004.docx]

Table S1. Baseline Characteristics of Redefined Study Subjects

|  | Normal  (n=13) | Low-ratio  (n=25) | High-ratio  (n=16) | *p*-value^*^ |
| --- | --- | --- | --- | --- |
| Age (years) | 75.6 ± 6.2 | 71.5 ± 7.4 | 74.3 ± 8.0 | 0.23 |
| Male | 10 (76.9%) | 17 (68.0%) | 8 (50.0%) | 0.29 |
| Education (years) | 16.8 ± 2.6 | 17.1 ± 2.1 | 14.5 ± 2.7 | < 0.01 |
| T^‡^ | a | a | b |  |
| MMSE | 28.6 ± 1.9 | 28.2 ± 1.4 | 27.4 ± 1.8 | 0.16 |
| ApoE ε4 carriers | 1 (7.7%) | 7 (28.0%) | 6 (37.5%) | 0.19^†^ |

Figures denote mean values and standard deviations. Parentheses indicate frequencies.

^*^ Statistical significances were tested by one-way analysis of variances among groups or Chi-square test.

^†^ Fisher’s Exact Test.

^‡^ The same letters indicate non-significant difference between groups based on Bonferroni multiple comparison tests.

Abbreviations: MMSE (Mini-Mental State Examination)
