# Supplementary material for: Differential White Matter Connectivity in Early Mild Cognitive Impairment According to CSF Biomarkers
Source: PLoS One. 2014 Mar 10;9(3):e91400. doi: 10.1371/journal.pone.0091400 (PMC3948821; doi:10.1371/journal.pone.0091400)
Supplement: Table S2 — CSF Biomarkers and Florbetapir Standard Uptake Value Ratios of Study Subjects. (DOCX) [file pone.0091400.s005.docx]

Table S2. CSF Biomarkers and Florbetapir Standard Uptake Value Ratios of Study Subjects

|  | Normal  (n=13) | Low-ratio  (n=25) | High-ratio  (n=16) | *p*-value^*^ |
| --- | --- | --- | --- | --- |
| CSF biomarkers |  |  |  |  |
| Aβ (pg/ml) | 291.46 ± 52.10 | 263.87 ± 52.07 | 171.71 ± 57.63 | < 0.01^†^ |
| pTau (pg/ml) | 18.31 ± 4.22 | 16.72 ± 3.49 | 35.63 ± 13.85 | < 0.01^†^ |
| Total Tau (pg/ml) | 63.81 ± 25.01 | 60.36 ± 20.39 | 125.44 ± 64.31 | < 0.01^†^ |
| pTau/Aβ ratio | 0.06 ± 0.02 | 0.07 ± 0.02 | 0.22 ± 0.11 | < 0.01^†^ |
| Florbetapir PET |  |  |  |  |
| Mean SUVR | 0.99 ± 0.06 | 1.02 ± 0.08 | 1.33 ± 0.21 | < 0.01^†^ |

Figures denote mean values and standard deviations.

^*^ Statistical significances were tested by one-way analysis of variance among groups

^†^ Bonferroni multiple comparison tests show significant differences between high-ratio vs. low-ratio or high-ratio vs. normal group. There are no significant differences between low-ratio and normal groups.

Abbreviations: CSF (cerebrospinal fluid), Aβ (amyloid beta_1-42_), pTau (phosphorylated Tau_181p_), PET (positron emission tomography), SUVR (standard uptake value ratio)
