# Supplementary material for: Differential White Matter Connectivity in Early Mild Cognitive Impairment According to CSF Biomarkers
Source: PLoS One. 2014 Mar 10;9(3):e91400. doi: 10.1371/journal.pone.0091400 (PMC3948821; doi:10.1371/journal.pone.0091400)
Supplement: Table S3 — Correlation coefficient (ρ) between CSF biomarkers and Florbetapir SUVR. (DOCX) [file pone.0091400.s006.docx]

Table S3. Correlation coefficient (ρ) between CSF biomarkers and Florbetapir SUVR

|  | Normal Control  (n=13) | EMCI | | |
| --- | --- | --- | --- | --- |
|  |  | Low-ratio  (n=25) | High-ratio  (n=16) | Total  (n=41) |
| pTau/Aβ ratio | -0.15 | 0.60^*^ | 0.58^†^ | 0.70^*^ |
| Aβ | 0.18 | -0.53^*^ | -0.28 | -0.80^*^ |
| Total Tau | 0.00 | 0.26 | 0.64^*^ | 0.59^*^ |
| pTau | 0.07 | 0.28 | 0.21 | 0.65^*^ |

Spearman’s correlation

^*^ p-value < 0.01, ^†^ p-value < 0.05
