# Supplementary material for: Differential White Matter Connectivity in Early Mild Cognitive Impairment According to CSF Biomarkers
Source: PLoS One. 2014 Mar 10;9(3):e91400. doi: 10.1371/journal.pone.0091400 (PMC3948821; doi:10.1371/journal.pone.0091400)
Supplement: File S1 — Supplemental Methods. (DOCX) [file pone.0091400.s007.docx]

**Supplemental Methods**

**Database**

ADNI was launched in 2003 by the National Institute on Aging (NIA), the National Institute of Biomedical Imaging and Bioengineering (NIBIB), the Food and Drug Administration (FDA), private pharmaceutical companies and non-profit organizations, as a $60 million, 5-year public-private partnership. The primary goal of ADNI has been to test whether serial magnetic resonance imaging (MRI), PET, other biological markers, and clinical and neuropsychological assessment can be combined to measure the progression of MCI and early AD. Determination of sensitive and specific markers of very early AD progression is intended to aid researchers and clinicians in developing new treatments and monitoring their effectiveness, as well as lessen the time and cost of clinical trials.

ADNI is the result of the efforts of many co-investigators from a broad range of academic institutions and private corporations; and subjects have been recruited from over 50 sites across the U.S. and Canada. The initial goal of ADNI was to recruit 800 subjects but ADNI has been followed by ADNI-GO and ADNI-2. To date these three protocols have recruited over 1500 adults, ages 55 to 90, to participate in the research, consisting of cognitively normal older individuals, people with early or late MCI, and people with early AD. The follow up duration of each group is specified in the protocols for ADNI-1, ADNI-2 and ADNI-GO. Subjects originally recruited for ADNI-1 and ADNI-GO had the option to be followed in ADNI-2. For up-to-date information, see [www.adni-info.org](http://www.adni-info.org).
